# A Comprehensive Review of the Pathophysiology of Neonatal Stroke and a Critique of Current and Future Therapeutic Strategies

**DOI:** 10.3390/cells14120910

**Published:** 2025-06-16

**Authors:** Victor Mondal, Emily Ross-Munro, Gayathri K. Balasuriya, Ritu Kumari, Md. Munnaf Hossen, Mohammed Ageeli, Kate Firipis, David R. Nisbet, Glenn F. King, Richard J. Williams, Pierre Gressens, Jeanie L. Y. Cheong, Flora Y. Wong, David W. Walker, Mary Tolcos, Bobbi Fleiss

**Affiliations:** 1 School of Health and Biomedical Sciences, RMIT University, Bundoora, VIC 3083, Australia; s3852042@student.rmit.edu.au (V.M.); emily.ross-munro@rmit.edu.au (E.R.-M.); gayathri.balasuriya@rmit.edu.au (G.K.B.); s4059311@student.rmit.edu.au (R.K.); s3986181@student.rmit.edu.au (M.M.H.); s3871305@student.rmit.edu.au (M.A.); dww1094@gmail.com (D.W.W.); mary.tolcos@rmit.edu.au (M.T.); 2 O’Brien Department, St Vincent’s Institute for Medical Research, Fitzroy, VIC 3065, Australia; kate.firipis@gmail.com; 3 Department of Medicine, St Vincent’s Hospital, Faculty of Medicine, Dentistry and Health Science, The University of Melbourne, Parkville, Melbourne, VIC 3010, Australia; 4 Aikenhead Centre for Medical Discovery, St Vincent’s Hospital, Fitzroy, VIC 3065, Australia; david.nisbet@unimelb.edu.au; 5 Department of Biomedical Engineering, Faculty of Engineering and Information Technology, The University of Melbourne, Parkville, VIC 3010, Australia; 6 The Graeme Clark Institute, The University of Melbourne, Parkville, VIC 3010, Australia; 7 Medical School, Faculty of Medicine, Dentistry and Health Science, The University of Melbourne, Parkville, VIC 3010, Australia; 8 Institute for Molecular Bioscience, The University of Queensland, St Lucia, QLD 4072, Australia; glenn.king@imb.uq.edu.au; 9 Australian Research Council Centre of Excellence for Innovations in Peptide and Protein Science, The University of Queensland, St Lucia, QLD 4072, Australia; 10 School of Medicine, Deakin University, Waurn Ponds, VIC 3216, Australia; richard.williams@deakin.edu.au; 11 Université Paris Cité, Inserm, NeuroDiderot, 75019 Paris, France; pierre.gressens@inserm.fr; 12 Clinical Sciences, Murdoch Children’s Research Institute, Parkville, VIC 3052, Australia; jeanie.cheong@mcri.edu.au; 13 Neonatal Services, Royal Women’s Hospital, Parkville, VIC 3052, Australia; 14 Department of Obstetrics, Gynaecology and Newborn Health, and Department of Paediatrics, University of Melbourne, Parkville, VIC 3052, Australia; 15 Monash Newborn, Monash Children’s Hospital, Clayton, VIC 3168, Australia; flora.wong@monash.edu; 16 Department of Paediatrics, Monash University, Clayton, VIC 3168, Australia; 17 The Ritchie Centre and Hudson Institute, Monash University, Clayton, VIC 3168, Australia

**Keywords:** regeneration, perinatal brain injury, biomedical engineering, hydrogel, cerebral palsy

## Abstract

Within the first 28 days after birth, more than 1 in every 2500 newborns will suffer a stroke. The weekly-adjusted risk of stroke for a term-born infant is threefold higher than for a male smoker aged 50 to 59 years with hypertension and diabetes. Neonatal stroke has significant clinical and socio-economic consequences, leading to cerebral palsy, epilepsy, and a range of motor, sensory, and cognitive impairments. Currently, there is no treatment for the brain damage caused by neonatal stroke. In this review, we outline the differences in the complex interplay of inflammation, excitotoxicity, oxidative stress, and cell death after stroke between adults and neonates, which limits the direct transfer of knowledge between studies for understanding injury. We comprehensively document what is known about the pathophysiology of neonatal stroke and critically evaluate current therapeutic strategies, emphasising the urgent need for innovative treatments tailored to suit the neonatal brain. This analysis reveals that treatment with an injectable hydrogel scaffold, a three-dimensional, water-swollen polymer network, may be an innovative, viable approach to improve outcomes for infants suffering from the most severe forms of brain injury arising from neonatal stroke.

## 1. Introduction

Globally, stroke across all ages is the second leading cause of death, and 50% of survivors are left with a lifelong disability. Although stroke is most commonly known to occur in older adults, babies in the first 28 days after birth have a weekly risk of stroke that is three times greater than that of a male aged 50 to 59 years who smokes, has diabetes mellitus, and has hypertension [1]. The diagnosis of stroke in babies is difficult, but in high-income settings with clear diagnostic pathways, rates are as high as 1 in 1100 live births [2,3,4,5,6]. In up to 50% of infants who develop a stroke early in life, it causes long-lasting impacts, including hemiplegic cerebral palsy, epilepsy, and cognitive, language, and behavioural deficits [7,8,9,10,11].

**Figure 1:**
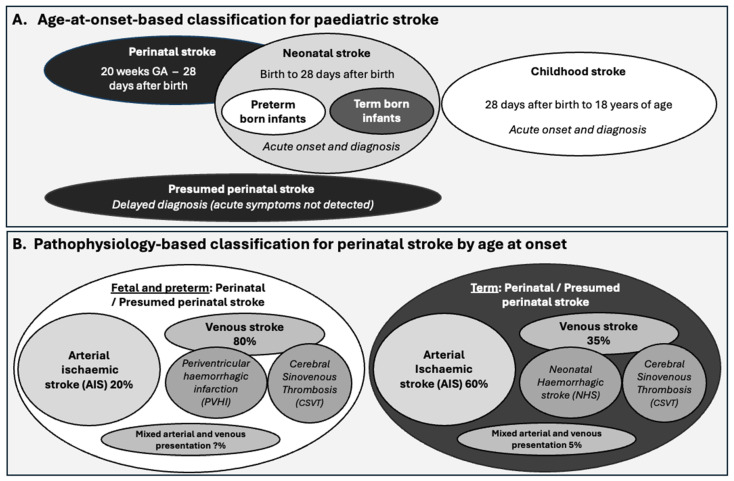
Outline of the (**A**) classification of paediatric stroke by age and (**B**) classifications within perinatal stroke based on pathophysiology. GA = gestational age.

The gestational or postnatal age at onset or diagnosis is initially used to describe the subtypes of paediatric stroke, including perinatal stroke (from mid gestation to 28 days after birth) and postnatal stroke (from 28 days after birth to 18 years of age) (Figure 1A). Stroke diagnosed a month or more after birth is termed a presumed perinatal stroke, with up to 50% of cases diagnosed in this period [12]. A delayed diagnosis is often precipitated when infants exhibit seizures for the first time weeks after birth or pathological early-handedness, prompting brain imaging studies that identify a remote infarction [13]. The stroke is then classified according to whether the underlying cause is ischemic or haemorrhagic, as detailed in the NIH Common Data Elements. It is worth noting that the type of stroke and the injury that arises differ substantially between foetal and preterm infants and term infant brains (Figure 1B) [14]. In this review, we focus on the pathophysiology of neonatal stroke (onset from birth to 28 days after birth) in term-born infants.

The most common symptom of perinatal stroke across term and also preterm-born infants is seizures, accompanied by altered cry, apnoea, extreme sleepiness, and lethargy [15]. These symptoms are challenging to recognise, are not specific to stroke, and diagnosis needs to be confirmed by magnetic resonance imaging (MRI). As such, the diagnosis of symptomatic stroke takes on average 22 h [16].

At this time, there are no therapies for the brain damage caused by perinatal stroke, although early intervention delivered by healthcare professionals, including physiotherapy and occupational therapy, is invaluable to minimise negative impacts [17,18]. Difficulties in the development of therapies for perinatal stroke have included the many decades of research focussed on acute phase treatments (to be initiated within the first 2–8 h), which have not been compatible with the diagnostic timing in this population. In addition, it is difficult to extrapolate knowledge directly from the adult stroke field as neonatal and adult brains have substantially different physiological and biochemical responses to injury [19]. Key differences between the adult and neonatal response to stroke are summarised in Table 1, and throughout the review, we highlight differences in responses to injury, and consequently, the therapeutic approach most likely to be most effective.

Thorough reviews of models of neonatal stroke and associated HI injuries have been published [20,21,22], so we only provide an overview of the key approaches in Table 2 to help orient our discussion of these models in the review. Of note is that there are few models of neonatal stroke in animals with large and gyrencephalic brains, limited to a model in the piglet [23] and the non-human primate [24].

**Table 1:** Summary of key differences between the adult and neonatal brain.

Characteristic	Difference	Ref.
*Tissue compliance “squishiness”*	The neonatal brain has a higher water content, less dense extracellular matrix, and lower myelin content, which confers higher compliance (‘squishiness’) and affects tissue elasticity.	[25,26]
*Oligodendrocyte development and myelination*	There is a greater vulnerability of oligodendrocyte precursor cells to injury and persisting dysmaturation.	[27,28]
*Inflammatory responses*	Neonates have lower neutrophil and lymphocyte infiltration and higher levels of anti-inflammatory proteins.	[29,30]
*Plasminogen system activation*	In neonates there is only transient fibrin deposition and impaired blood perfusion and acute induction of both tissue-type and urinary-type plasminogen activators (tPA and uPA).	[31]
*Blood–brain barrier function*	There is less stroke-induced permeability in neonatal models of stroke.	[29]
*Injury pattern/subtype*	Neonates have haemorrhagic stroke more often than adults. In a small animal model, neonate haemorrhages were superficial in the cortex; however, in adults they were larger and in the cerebellum.	[32]
*Cell death process*	The neonatal brain undergoes caspase-3-mediated cell death more than the adult brain. There are also higher levels of delayed phase apoptosis in the neonate than the adult.	[33,34]
*ROS accumulation*	Neonates have lower levels of antioxidants such as metallothionein I and II in the brain than adults.	[34,35,36]
*Sex differences*	Sex differences are more apparent in neonates. Neonatal males are more severely affected. In adulthood, females are more affected.	[37,38,39,40]

**Table 2:** A summary of key models of neonatal stroke.

Induction Method	Species Applied in	Description	Pros	Cons	Clinical Phenotype Links	Selected Ref.
**PT Stroke**	Mouse, rat, and piglet	Induced via systemic injection of a photosensitive dye (e.g., Rose Bengal) followed by targeted light exposure, leading to localized infarction.	- Highly reproducible infarcts - Precise lesion localization - Non-invasive (no craniotomy required) - Suitable for animals of different sizes	- Mostly cortical infarcts (limited subcortical involvement) - Lacks penumbra-like region (less critical in neonates where there are diagnostic delays) - Induces endothelial damage via photoactivation, not vascular occlusion	Mimics focal cortical infarcts seen in some AIS, though mechanism differs from thromboembolic causes.	- P7 rat [41,42] or P7 mouse, requiring scalp incision. Motor cortex lesion - P10 rat, requiring scalp incision. Motor cortex lesion [43] - P17 piglet, requiring craniotomy and dural thinning. MCA specific [23]
**ET-1 Injection**	Mouse, rat, and non-human primate	Focal vasoconstriction achieved by microinjection of ET-1 near cerebral arteries, resulting in transient or permanent ischemia.	- Induces vasoconstriction without direct vessel occlusion - Adjustable infarct size - Allows for studies on reperfusion injury	- Variability in infarct size - Potential for ET-1 diffusion beyond target site - Not a natural stroke mechanism (pharmacological induction)	Models transient focal ischemia and vasospasm; mimics elements of hypoperfusion and reperfusion injury in neonates.	- P0 rat. Striatal and cortical lesion [44] - P14 marmoset requiring craniotomy and dura thinning. PCA specific [45]
**Embolic Stroke Model**	Mouse	Injection of autologous blood clots or synthetic microspheres into the carotid artery to induce embolic occlusion of cerebral vessels.	- Mimics natural thromboembolic events - Relevance to clinical AIS pathophysiology - Allows for thrombolysis studies	- Technically challenging in neonates - Variability in embolus size/location - Risk of multi-territory infarcts - Less reproducible	Closest to presumed perinatal AIS of embolic origins (e.g., from placenta or cardiac defects).	- Magnetised RBC induced embolism in P5–7 mice [46] - No other studies found
**Middle Cerebral Artery Occlusion (MCAO)**	Mouse, rat	Intraluminal filament inserted via the external carotid artery to occlude the MCA, modelling both permanent and transient ischemia.	- Widely used, well-characterized - Can model both transient and permanent ischemia - Mimics large territorial infarcts - Penumbra formation	- Invasive; technically challenging in neonates - Risk of temperature/hypoxia confounders - Requires precise microsurgical skills	Closely mimics neonatal AIS in the MCA territory (most common in term infants); supports study of infarct evolution and therapies.	- P7 rat [47] permanent left MCA electrocoagulation with transient left CCA occlusion - P9 mouse [48] and rat [49] transient MCAO - P9 mouse [50] permanent via filament - P12 mouse [51] permanent via electrocoagulation
**Hypoxia-Ischemia (Rice-Vannucci Model)**	Mouse, rat	Unilateral common carotid artery ligation then exposure to hypoxic conditions; widely used to model perinatal HIE.	- Widely used - Simple to perform in neonatal rodents - Produces large unilateral brain injury including to the cortex, striatum, and hippocampus	- Not representative of neonatal AIS - Global hypoxic insult + unilateral carotid ligation - Lacks thromboembolic or focal vascular mechanism - Diffuse injury pattern - Alters systemic physiology	Models HIE, not AIS; misapplication leads to misleading translational conclusions.	Comparison of territories effected in MCAO and HIE-models [52]

**Abbreviations**: AIS, arterial ischemic stroke. CCA, common carotid artery. HIE, hypoxic-ischemic encephalopathy. ET-1, Endothelin-1. PCA, posterior cerebral artery. PT, photothrombotic. RBC, red blood cell.

## 2. Types of Perinatal Stroke, Areas Affected, and Contributing Factors

The causes of perinatal stroke are classified as ischemic—including an arterial ischemic event (impaired blood entry), and, less commonly, cerebral venous sinus thrombosis (impaired blood exit)—or haemorrhagic (bleeding), including intraventricular, intraparenchymal, or subarachnoid haemorrhage (Figure 1B) [1]. The incidence of intraventricular haemorrhage with periventricular haemorrhagic infarction is higher in infants with lower gestational age due to the immaturity of the ventricular vascular bed. It is less common in term neonates, typically secondary to haemorrhage near the ventricle, and is regarded as a separate clinical entity [53].

Arterial ischemic stroke (AIS) accounts for ~60% of perinatal strokes in term-born infants, with an incidence that varies from 1 in 2500 to 1 in 7500 live births, depending on the diagnostic criteria and clinical surveillance [1,54,55,56]. Vessels typically affected in neonatal AIS (NAIS) include the middle cerebral artery (MCA) [1,57], thalamic branches of the posterior cerebral artery, and the perforating arteries [58,59]. Table 3 summarises the major arteries most commonly affected and the impacts of stroke in these areas on neurological function. Across all forms of neonatal stroke (arterial, haemorrhagic, and CSVT), due to the prevalence of involvement of the MCA, damage to the lateral surfaces of the frontal, parietal, and temporal lobes is found in 60–70% of cases. Combined involvement of the basal ganglia and thalamus is reported in 30–40% of cases. Cerebellar involvement is reported in approximately 10–15% of cases, and even less common is the involvement of the occipital lobe. Lesion volumes in neonatal stroke average 10 mm^3^ in the neonatal period and expand to 20 mm^3^ by young adulthood. Additionally, changes such as reduced cortical thickness and connectivity are observed in the contralateral hemisphere [60,61], indicating long-range impacts on brain development. 

Intrapartum, maternal, and foetal factors contribute to the development of perinatal stroke, although their interactions are not fully understood [3,68,69]. Intrapartum factors associated with an increased risk for NAIS specifically include a low Apgar score, emergency caesarean section, resuscitation at birth, abnormal foetal heart rate, prolonged labour (particularly the second stage), and meconium liquor. Birth trauma itself is no longer considered a cause of NAIS, although exposure to hypoxia-ischaemia (HI) around the time of birth is an additive risk factor [3]. Maternal/placental risk factors include nulliparity, intrauterine growth restriction, and foetal vascular malperfusion. Foetal vascular malperfusion refers to a group of placental lesions that indicate reduced or absent perfusion of the villous parenchyma by the foetus [70]. Obstruction of the umbilical cord is the most common cause of malperfusion, resulting in stasis, ischemia, and occasionally thrombosis. There is also a strong correlation between an increased risk for NAIS and placental thromboembolism, chorioamnionitis, and congenital heart disease [13]. Modest risk factors for NAIS include preeclampsia, maternal prothrombotic disorders, and bacterial meningitis [71]. Research has not found a consistent link with inherited prothrombotic factors [72]. Males are more commonly affected by all forms of stroke, including NAIS, compared with females, and the outcomes for males in most forms of hypoxic-ischemic injury are worse than for females. Kelly and colleagues [40] recently reviewed the role of sex in perinatal brain injury, which we discuss further below as it relates to neonatal stroke.

## 3. The Pathophysiology of Term Infant NAIS

**Figure 2:**
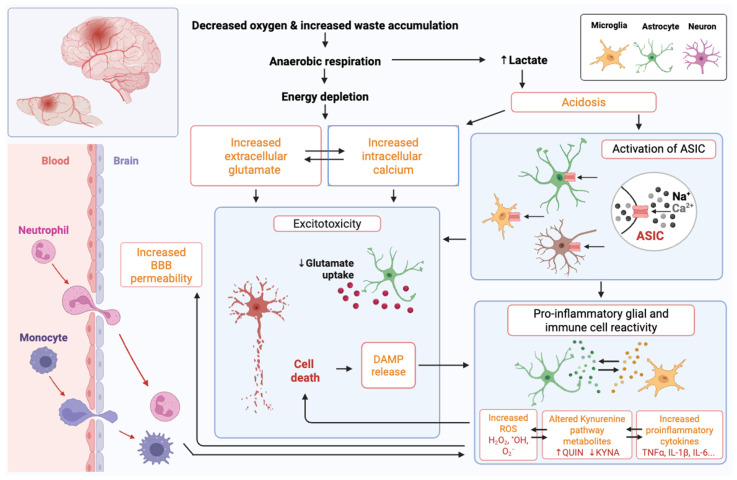
Overview of events following neonatal stroke with a focus on the mechanisms that drive cell death and pro-inflammatory cellular reactivity. Abbreviations: Ca^2+^, calcium; ASIC, acid-sensing ion channel; BBB, blood–brain barrier; DAMP, damage-associated molecular pattern; H_2_O_2_, hydrogen peroxide; *OH, hydroxyl radical; O_2_^−^, superoxide; QUIN, quinolinic acid; KYNA, kynurenine; TNF-α, tumour necrosis factor-alpha; IL, interleukin.

The primary stages and the processes leading to tissue injury after an arterial ischemic event are similar in neonates and adults [73], as outlined in Figure 2. Ischemic stroke disrupts energy metabolism, which contributes to cell death caused by glutamate excitotoxicity, acidosis, oxidative stress, and neuroinflammation. Blood flow decreases primarily in the infarct core, resulting in a severe disruption of energy production. However, these processes also cause damage in the peri-infarct region (penumbra), which then progresses over the following hours to days. Restoring blood flow to reduce injury severity is the central tenet of adult stroke therapy design, as the event onset is typically easy to identify. Thus, working to restore blood flow quickly to protect this tissue through catheter-based interventions, such as removing the clot and using thrombolytics to enzymatically degrade it, has proven effective in adults. However, due to the delay in diagnosis in neonates, which is typically many hours or even a day or more, it is not feasible to focus on the restoration of blood flow, and the interventions that work in adults (clot removal and thrombolytics) are of high risk and not established in small infants [74]. In the future, improved routine, low-cost bedside imaging, such as ultrafast Doppler [73,75], low-field portable magnetic resonance imaging [76], and miniaturised computed tomography [77], may provide accessible monitoring and assessment of blood flow that decreases time to diagnosis, creating opportunities for targeted approaches to restore blood flow. The numerous age-dependent differences in injury responses (Table 1), such as the nature of the immune response, contributing cell types, and cell-type-specific vulnerability to injury, also provide unique therapeutic opportunities.

### 3.1. Metabolic Failure, Excitotoxicity, and Cerebral Perfusion Anomalies

Restricted supply of nutrients and oxygen due to ischemia prevents ATP production, leading to the failure of sodium–potassium and calcium pumps and increased intracellular calcium concentration [74,78]. Elevated intracellular calcium induces glutamate release into the synaptic cleft by activating proteins such as synaptotagmin, which then leads to exocytosis of synaptic vesicles [79]. This triggers an excitotoxic cascade, in which glutamate leads to neuronal depolarisation by opening *N*-methyl-D-aspartate (NMDA) and α-amino-3-hydroxy-5-methyl-4-isoxazolepropionic acid (AMPA) receptors, leading to further calcium influx [76]. The dysregulation of ATP-dependent calcium channels activates lipases, proteases, and DNases, causing mitochondrial damage and further energy depletion [77]. In neonatal infants and rodents, γ-aminobutyric acid (GABA) also has excitatory properties in the early postnatal period [80,81]. Interestingly, there is a higher expression of AMPA receptors in the developing brain than in adults [82]. However, AMPA and NMDA receptor subunit composition differences render immature neurons less sensitive to glutamate-mediated excitotoxicity [83].

Non-neuronal cells, including oligodendrocytes and microglia, also express glutamate receptors [84,85]. NMDA receptor activation in oligodendrocytes increases the expression of glucose transporter type 1 [85]. Observations on glutamate and glucose signalling in oligodendrocytes led to the hypothesis that NMDA receptor activation regulates energy availability in oligodendrocytes, causing active axons to be myelinated preferentially [86]. In vitro, the activation of glutamate receptors on oligodendrocytes causes oligodendrocyte death, and microglial glutamate receptor activation induces a pro-inflammatory immune response, with elevated inducible nitric oxide synthase (iNOS) contributing to neuronal cell death [84].

Oligodendrocytes also express non-NMDA type glutamate receptors activated by kainate and AMPA that are Ca^2+^ impermeable but mediate the entry of monovalent ions such as Na^+^ and K^+^ [87]. An influx of Ca^2+^ through non-NMDA glutamate receptors triggers cell death in immature oligodendrocytes. Driving elevated Ca^2+^ is an autologous feedback loop, wherein immature oligodendrocytes release glutamate via reverse glutamate transport. Fern & Möller (2000) [88] found that cultured immature oligodendrocytes (O4+/galactoceramide−) are highly susceptible to ischemic injury, with 80% of cells dying after 25 min of oxygen and glucose deprivation compared to mature oligodendrocytes (O4+ galactoceramide+), where the same conditions killed only 43% of the mature cells after 42 min.

Following the initial ischemic injury, restoring blood flow, known as reperfusion, leads to a surge in the production of reactive oxygen species (ROS) (see the Oxidative Stress section below). This affects mitochondrial function and exacerbates inflammation and injury, a process called “reperfusion injury” [89]. Reperfusion is necessary, as ongoing lack of blood flow causes even greater injury, but managing this process is key in stroke management. The delay in the diagnosis of neonatal stroke makes the application of therapies that would induce reperfusion unrealistic, including endovascular therapy (physical clot removal) and clot-dissolving therapy (i.e., the administration of tissue-type plasminogen activator, tPA), which need to be applied within 3–4 h of stroke onset. Additionally, among neonatologists, including neurovascular surgeons, there are uncertainties regarding the stability of cerebral blood vessels and also of the potential toxicity of thrombolytic agents in infants. Hence, the utility of endovascular therapy is less evident in neonates than in adults [90]. Collectively, these factors mean that the focus of studies on the development of neurotherapeutics for neonatal stroke are less on preventing reperfusion injury than on promoting the regeneration of lost tissue.

Studies using magnetic resonance perfusion imaging in term newborns diagnosed with perinatal stroke within the territory of the right and left MCA show that brain perfusion abnormalities occur in the infarcted area during the acute phase (i.e., within 72 h of birth), which then persist into the subacute phase (5 to 6 days after birth). These abnormalities include hypoperfusion within the stroke territory and hyperperfusion at its periphery [91,92]. Hypoperfusion is associated with decreased metabolic demands following an irreversible brain injury, whereas persisting hyperperfusion may indicate a delayed recovery of cerebral blood flow autoregulation or an ongoing response mechanism to injury [93,94], including the potential for hyperoxia-induced oxidative stress. Increased vascular density with newly formed capillaries was observed around and after seven days post-stroke in a cohort of infants [65].

### 3.2. Oxidative Stress

Free radicals are atoms or molecules with an unpaired electron, such as superoxide (O_2_^−^), hydrogen peroxide (H_2_O_2_), hydroxyl radical (•OH), and singlet oxygen (O_2_). These are by-products of normal metabolic processes and serve roles in signalling and immune responses [95]. Mitochondria, often called ‘the powerhouses of the cell’, generate most ROS. Microglia and astrocytes also express ROS-producing NADPH oxidases (NOX) located in the cytosol, nucleus, endosome, endoplasmic reticulum, and lysosomal membranes [96,97]. Oxidative stress occurs when there is an imbalance between the production of free radicals and their elimination by antioxidant defences [98]. The body uses enzymes such as superoxide dismutase (SOD) and glutathione peroxidase (GPX), along with molecules like vitamins C and D, to neutralise free radicals. Oxidative stress damages DNA, proteins, and lipids, induces malondialdehyde production, causes inflammation, and ultimately results in the death of the cell [99]. Vitamin D, GPX, and SOD levels from venous blood samples were significantly lower on the first day of life in 31-term neonates following HI injury compared to 30 healthy-term neonates. At the same time, malondialdehyde levels were significantly higher in the HI group, indicating less effective antioxidant processes in the injured newborn brain [100].

Rapid perfusion induces ROS production, causing oxidative stress [101]. Free radical production is high whenever oxygen levels increase quickly, such as during rapid reperfusion [102]; for example, after tPA-induced reperfusion and mechanical thrombectomy, which is the standard of care for adult stroke patients. Oxidative stress is more pronounced after neonatal stroke than adult stroke, not only for the reasons given above but also due to higher oxygen consumption in the neonatal brain, a higher concentration of unsaturated fatty acids, and a lower concentration of antioxidants [103,104]. Notably, oligodendrocyte precursor cells are particularly vulnerable to oxidative stress [105,106]. As a result of free radical formation and excitotoxic injury, pre-oligodendrocytes die or their maturation is disrupted [107]. Specifically, oxidative stress decreases the expression of differentiation-promoting genes such as *Olig1* and *Sox10* in pre-oligodendrocytes while increasing the expression of differentiation-inhibiting genes such as *ID2* and *ID4*, ultimately interrupting oligodendrocyte maturation [108]. Microglia exhibit robust responses in models of HI, including increased production of ROS [109] and release of ROS-inducing proteins such as galectin-3 [110]. Neurons in the immature brain produce higher amounts of ROS in response to stress; for instance, immature versus mature cultured cerebellar neurons generate more ROS when exposed to an acetylcholinesterase inhibitor [105].

### 3.3. Acidosis

Brain pH is tightly regulated at 7.3 extracellularly and 7.0 intracellularly [111,112]. HI causes acidosis, such that pH in the ischemic core can decline to 6.5 in normoglycemic rats, but in hyperglycaemic rats it can fall further to 6.0 [113,114]. Cerebral acidosis occurs in infants after neonatal stroke and systemic HI injury and can persist for up to 30 weeks post-insult [115,116]. Hypothermia protects against some forms of ischemic injury, mediated partly by a reduction in acidosis [117].

Arachidonic acid (AA) levels are increased for many days post-ischemia due to the phospholipase A2-mediated degradation of membrane lipids [118,119]. The cyclooxygenase enzyme COX2 converts AA to immune-reactive prostaglandins and neurotoxic lipoxygenase 2. AA also affects voltage-gated and ligand-gated ion channels, including NMDA receptors and acid-sensing ion channel 1a (ASIC1a), which are proton-gated sodium ion channels activated by a tissue pH below 7.0 [120,121]. There are six ASIC subunits, with ASIC1a predominant in human and rodent brains [122,123,124]. Homomeric ASIC1a, ASIC1b, and ASIC3 channels are sodium-selective, whereas ASIC1a also has significant calcium permeability [125]. AA potentiates ASIC-dependent proton-gated currents in cultured rat dorsal root ganglion or cerebellar neurons [126]. ASIC1a knockout reduces brain injury in an adult mouse MCAO model, while in vitro studies verified that ASIC1a depletion protected the brain by reducing calcium influx in a glutamate-independent manner [125]. Robust decreases in the expression of ASIC1a mediate the neuroprotective effects of pre- and post-hypoxic conditioning in a mouse adult stroke model [127].

In neonatal mice with brain injury, bilirubin-induced neurotoxicity is associated with acidosis and ASIC1a activation, with injury not observed in ASIC1a knockout neurons [128]. In a piglet model of term infant asphyxia-induced cardiac arrest, pre-infusion into the lateral cerebral ventricle (but not post-injury infusion) with a highly selective ASIC1a inhibitor, psalmotoxin-1 (PcTx1), significantly reduced peroxynitrite-mediated protein damage and neuronal loss [129]. Neuroprotection has also been observed with amiloride, a non-specific ASIC inhibitor, with pre-treatment in a neonatal mouse model of HI injury [130], post-treatment in a piglet model of HI injury [131], and combined with hypothermia in a neonatal rat brain slice model of HI injury [132].

Peptide Hi1a from Australian funnel-web spider *Hadronyche infensa* venom is the most potent and selective inhibitor of ASIC1a described to date [133]. In an MCAO model of adult stroke in spontaneously hypertensive rats, induced by endothelin injection over the MCA, Hi1a improved functional and neurological outcomes, providing neuroprotection to the penumbra as well as to the core of the infarct, which is generally considered ‘unsalvageable’ [133]. Interestingly, intracerebral administration of Hi1a afforded a similar level of neuroprotection when administered at 2, 4, or 8 h post-stroke [133]. When administered via intravenous injection, Hi1a protected against sensorimotor deficits and reduced striatal, but not total, lesion volume in an adult rat endothelin-induced stroke model, likely due to limited penetration across the blood–brain barrier (BBB) [134]. In neonatal stroke, ASIC targeting is of particular interest, because acidosis, as measured using magnetic resonance spectroscopy, persists for many months after a neonatal HI injury in infants [116], as do the processes of cell death in models of HI injury [135,136].

### 3.4. Cell Death

Only a few groups have studied models of neonatal stroke where there is an ongoing obstruction of blood flow, such as direct MCA occlusion (MCAO), or vascular occlusion via photothrombotic damage or vasoconstriction. There have been very few post-mortem studies, so data are scarce on patterns and types of cell death. Generally, across studies of HI and stroke, the components of injury that induce cell death and brain damage—excitotoxicity, oxidative stress, and inflammation—are similar [137]. Therefore, we have also included studies on HI injury, most notably the Rice–Vannucci model, which combines carotid artery ligation and global hypoxia. This model more accurately mimics HI encephalopathy (HIE), involving the restriction in blood flow and oxygenation because of umbilical cord occlusion or placental abruption, leading to a global insult with injury patterns often including the thalamus, basal ganglia, and the posterior limb of the internal capsule, plus watershed regions between major cerebral arteries [52]. Regions impacted by stroke are more likely to be focal, including in the cortex, basal ganglia, and periventricular white matter. The nature of the injury, global versus focal, the duration of the insult, and any spontaneous reperfusion events all influence the severity of the insult and will impact the type of cell death.

**Figure 3:**
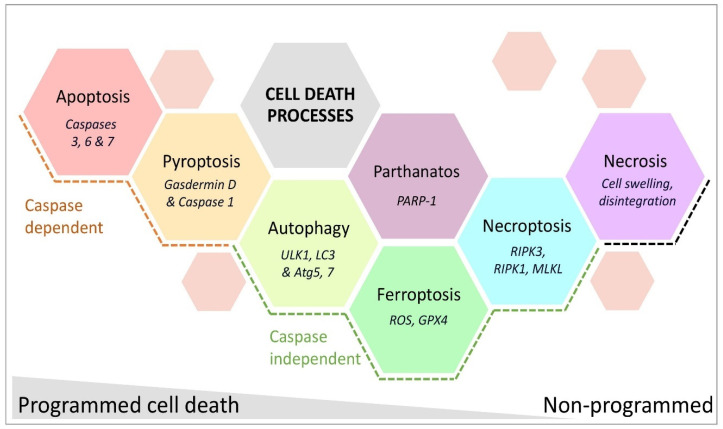
Cell death processes observed after neonatal stroke categorised as programmed or non-programmed subtypes. Molecules characteristic of each process are in italics. Abbreviations: ULK1, unc-51 like autophagy activating kinase 1; LC3, microtubule-associated protein 1A/1B light chain 3B; Atg, autophagy-related; ROS, reactive oxygen species; GPX4, glutathione peroxidase 4; PARP-1, poly [ADP-ribose] polymerase 1; RIPK, receptor-interacting protein kinase; MLKL, mixed lineage kinase domain-like protein.

Cell death due to HI injury in the developing brain manifests in the necrotic–apoptotic continuum [138,139,140], with apoptosis and necrosis observed in the same regions after injury [141,142,143]. In models of adult and neonatal stroke induced with MCAO [144,145], cell death becomes persists for weeks after injury, suggesting that treatments for cell death may be effective in the tertiary phase [146]. Here, we briefly describe the various forms of cell death and their known roles in HIE and neonatal stroke and the processes are summarised in Figure 3.

#### 3.4.1. Caspase-Dependent Programmed Cell Death—Apoptosis and Pyroptosis

Apoptosis in neonates with HI-associated brain injury occurs via intrinsic or extrinsic pathways [147]. Mitochondrial membrane permeabilisation releases cytochrome c and other apoptogenic factors. This triggers caspase-dependent cell death via the initiator caspase 9, activating the effector caspases 3, 6, and 7 [148]. The mitochondria-derived protein signals, endonuclease G, and apoptosis-inducing factor induce caspase-independent cell death [149]. The extrinsic apoptotic pathway, driven by the activation of death receptors, involves initiator caspases 8 and 10. Compared to the adult brain, higher levels of apoptosis are present in the developing brain, as caspases play critical roles in programmed or developmental cell death [33]. Apoptosis is the main form of cell death after MCAO in postnatal day (P) 7 rats [150] and also after ischemia with reperfusion (unilateral carotid artery ligation and short-term hypoxia) in P9 mice [151]. Pan-caspase inhibitors such as Q-VD-OPh provide greater neuroprotection to female P7 rats in MCAO-induced injury, as females express greater levels of activated (cleaved) caspase 3 post-stroke than males [152]. Females continue to express higher levels of caspases (vs males) into adulthood in response to an MCAO-induced injury [153].

Pyroptosis, a relatively recently characterised type of cell death linked to sepsis, inflammatory diseases, and HI insults [154,155], occurs through hypoxia-inducible factor 1α-dependent activation of the NLR family pyrin domain containing 3 (NLRP3) inflammasome complex, which includes caspase 1 [156]. This process triggers gasdermin D-dependent release of cytokines from microglia [157] and other cells [142]. Elevated levels of pyroptosis-associated proteins (NLRP3, gasdermin D, caspase 1, and interleukin [IL]-1β) in infants diagnosed with HIE correlates with insult severity [158]. Studies in P7 rats subjected to carotid artery ligation + hypoxia, and microglia cultures under oxygen-glucose deprivation, highlight NLRP3-driven pyroptosis in microglia. In 10-day-old rats subjected to carotid artery ligation + hypoxia, neuronal pyroptosis links to endoplasmic stress, dependent on increased expression of microRNA-125 and NLRP, providing a new therapeutic target [159].

#### 3.4.2. Caspase-Independent Programmed Cell Death—Autophagy, Necroptosis, Ferroptosis, and Parthanatos

Autophagy, the process of self-degradation to recycle cellular components, is vital for clearing damaged or redundant organelles and cells. Double-layered autophagosome vesicles engulf damaged organelles and proteins and then fuse with lysosomes, enabling their degradation to provide resources for energy and protein synthesis [160]. It has not been specifically shown whether autophagy is higher in the neonatal versus the adult brain. However, the critical role of autophagy in development is highlighted by the fact that ablation of key autophagy genes causes embryonic lethality in mice [161], and studies of aging [162] show that autophagy declines as the risk of neurodegenerative diseases increases. This suggests that autophagy may be a more significant and dominant process in the immature brain compared to the adult brain.

Supporting that autophagy could be a more important and dominant process in the immature versus the adult brain is that in a neonatal model of MCAO in P12 rats, hypoxic injury increased lysosomal activity and autophagosome formation, indicative of enhanced autophagy. This increased autophagy occurred primarily at the lesion border and contributed to delayed cell death. Intracerebroventricular administration of the autophagy inhibitor 3-methyladenine at four hours post-MACO reduced lesion volume by 46% [163]. Similarly, in a mouse model of severe HI (P9, carotid artery ligation + hypoxia), deletion of autophagy protein 7 significantly protected the brain, reducing lesion volume by 40% [164]. These findings highlight autophagy as a potential therapeutic target following neonatal stroke.

Necroptosis combines apoptotic signalling with a necrotic cell-death phenotype. It involves the formation of the necrosome by receptor-interacting protein kinase 1 (RIPK1), RIPK3, and mixed lineage kinase domain-like protein (MLKL) [165,166]. This process is prevalent in the stroke core and penumbra for hours to days following HI insults in neonatal models. In P7 rats, necroptosis appeared prominently in severely injured brain areas [138,139,167], while in P11 mice, necrosis dominated the cell death phenotype [139]. Activation of acid-sensing ion channel (ASIC)-1a leads to the recruitment and phosphorylation of RIPK1, thus inducing necroptosis [168]. Blocking necroptosis with Necrostatin-1 provides neuroprotection in neonatal HI injury models, specifically in rats and mice pups where brain injury is induced via carotid artery occlusion and hypoxia [169,170]. Inflammation, particularly production of the classic inflammatory mediator TNF-α, is the primary molecular driver of necrosome formation, as illustrated in an adult rat transient MCAO model, where TNF-α induced endothelial cell necroptosis led to BBB breakdown [171]. Blocking necroptosis prevented BBB breakdown and reduced brain injury [169,170]. The role of the BBB in neonatal stroke is detailed in a section below.

Ferroptosis, a form of programmed cell death, involves the accumulation of iron-dependent lipid peroxidation products and increased lipoxygenase and nicotinamide adenine dinucleotide phosphate (NADPH) oxidase [172], which is also induced by ROS-mediated cell membrane damage. Ferroptosis is affected by ferroprotein, lipoxygenase, and NADPH [172]. Ferroptosis plays a role in cell death in neonatal stroke in infants and neonatal animal models [173,174,175,176]. Clausi et al. [177] tested the efficacy of intranasal delivery of apotransferrin, which binds free ferric ions (Fe^3+^) and prevents uncontrolled tissue accumulation of iron, in a P10 mouse model of MCAO. This treatment reduced white matter damage and promoted myelin repair. Ferroptosis is a promising therapeutic target for neonatal stroke, but since most research has focused on adult stroke, the safety and efficacy of ferroptosis inhibitors in neonates require further investigation.

Parthanatos, mediated by poly (ADP-ribose) polymerase-1 (PARP-1) activation, is a caspase-independent, programmed cell death pathway [178]. This pathway exhibits distinct morphological and biochemical characteristics, including DNA damage, mitochondrial dysfunction, and the translocation of apoptosis-inducing factor from the mitochondria to the nucleus. HI induces PARP-1 activation to repair damaged DNA, but overactivation leads to NAD^+^ depletion and ATP starvation, resulting in cell death [179,180]. PARP-1 knockout in mice resulted in smaller infarcts and better neurological outcomes after stroke induced by MCAO [181]. In 7-day-old mice, a stroke lesion induced by MCAO activated PARP-1 in a sexually dimorphic manner, and PARP-1 gene deletion only protected males [182]. Similarly, in the amygdala of male but not female mice subjected to MCAO at P9, PARP inhibition decreased the number of cells positive for both ionised calcium binding adaptor molecule 1 (IBA1) and cyclooxygenase 2 (‘M1-like pro-inflammatory’; see microglia section below) [38]. PARP-1 inhibition also reduced apoptosis-inducing factor nuclear translocation and cell death in an adult rat model of MCAO stroke [183,184].

#### 3.4.3. Uncontrolled Cell Death—Necrosis

Necrosis, a form of uncontrolled or ‘accidental’ cell death, results in the disintegration of cells from within and this begins almost immediately in response to severe ATP depletion. Within 30 min, it can cause cell lysis due to membrane dysfunction and swelling in adult and neonatal models of HI injury [147]. Ca^2+^-dependent activated calpains are suggested markers of necrotic cell death, but no well-defined protein markers exist. Necrosis is more reliably identified by its morphological impacts on the cell in the early phases [185]. Puyal and colleagues investigated necrosis following neonatal cerebral ischemia in a P12 Sprague-Dawley rat MCAO model [163]. Western blot analysis revealed a rapid, substantial (280–640-fold), and sustained increase in calpain after ischemia, and electron microscopy of the centre of the lesion showed neurons with necrotic morphology.

### 3.5. Neuroinflammation

Neuroinflammation is a complex process characterised by the immune activation of microglia and astrocytes following most neurological injuries [186,187,188]. Neuroinflammation includes cytokine, chemokine, and ROS production, which damage surrounding cells, alter BBB integrity, and recruit other immune cells, such as macrophages and neutrophils, further enhancing inflammation [189,190,191]. Neuroinflammation is sufficient to cause cell death or dysmaturation [192,193], and a genetic predisposition to a pro-inflammatory immune response can lead to poorer neurological outcomes [194]. Comparing the adult and the neonate immune response, the neonate has been described to have an exaggerated [195] or unique [196] response in vivo, but whether these differences indicate vulnerability is unknown due to the complex in vivo processes.

#### 3.5.1. Microglia, Macrophage, and Monocytes

Macrophages are tissue-resident monocytes with tissue-specific functional repertoires [197]. Microglia are central nervous system (CNS) residents closely related to macrophages, with a unique embryonic lineage (yolk sac versus bone marrow-derived) [198], and are locally self-renewing [199]. Markers such as IBA1, cluster of differentiation (CD) 11b, or major histocompatibility complex II (MHCII) identify microglia, monocytes, macrophages, and other lymphocytes. Microglia-specific markers include the purinergic receptor P2RY12, splat-like transcription factor 1 (SPALT1), and the lysosomal enzymeβ hexosaminidase B (HEXB) [200]. Microglia regulate processes of normal brain development, such as neuronal migration, synaptic pruning, and neuronal and oligodendrocyte maturation [201,202,203]. Microglia progress through distinct stages of functional maturation regulated by chromatin remodelling, ensuring their abilities are matched to their roles in brain development [204]. For instance, between E15 and P45 in rodents (considered equivalent to the final trimester to adolescence in humans), microglia modulate functional connectivity via synaptic pruning and the release of trophic factors such as insulin-like growth factor 1 (IGF1) and brain-derived neurotrophic factor (BDNF) [205]. The functions of microglia evolve with age to focus primarily on injury surveillance, neuronal circuit function, and the maintenance of homeostasis, such as, for instance, via βamyloid clearance [206]. Reducing the adverse effects of injury-induced microglial responses while enabling microglia to fulfil these developmental roles must be fully considered in the search for microglia-mediating neurotherapeutics.

The simplest description of the response of microglia to injury or inflammatory stimuli is the M1 pro-inflammatory and M2 anti-inflammatory nomenclature; this paradigm incorrectly assumes that all microglia are identical and thus respond the same way to a challenge, and that there are only two response states. This nomenclature has fallen out of favour [77] because regional and temporally regulated subpopulations of microglia have specific and different impacts and outcomes [207,208]. Nevertheless, to communicate ideas and as a starting point, we can generalise that microglia in the adult and neonatal brain respond to insults (hypoxia, low ATP, high glutamate), damage-associated molecular patterns (DAMPs), such as intracellular proteins and DNA, or pathogen-associated molecular patterns (PAMPS) by expressing markers associated with a pro-inflammatory immune state, such as chemoattractants and ROS. These responses aim to destroy pathogens and remove debris after insults such as stroke. Generally, over time after injury, microglia as a population transition to expressing higher levels of markers associated with immuno-regulatory and anti-inflammatory functions, including the production of neurotrophins such as insulin-like growth factor 1 (IGF1) that support repair and regeneration [209,210,211,212]. The elevated expression of markers associated with pro-inflammatory responses is necessary for microglia to transition to a repair-process-oriented phenotype [213]. Post-stroke, neonatal microglia react and proliferate more rapidly than adult microglia [214]. Microglia initially form a border around the damaged tissue and gradually infiltrate the lesion to phagocytose cellular debris, enhancing the expression of phagocytic antigens such as CD11b and CD68 [215,216].

The Vexler group has demonstrated significant roles for microglia in neonatal stroke by differentiating them from macrophages and using microglial ablation. In their transient MCAO model in a P7 neonatal rat, they observed that microglia (CD45^low^/^medium^/CD11b^+^) accumulated in the first 24 h after injury [214]. Ablating microglia with clodronate liposomes before stroke onset significantly worsened the parenchymal inflammatory milieu and increased lesion volume 72 h post-injury [217]. Higher haemorrhagic occurrence was linked to elevated TGF-β signalling in the absence of microglia [218]. Similarly, in an adult MCAO stroke model, removing microglia via chronic treatment with the CSF1R antagonist PLX3397 increased stroke lesion volume and worsened functional deficits [219].

The Charriaut-Marlangue lab has also significantly advanced our understanding of microglia in neonatal stroke using transient MCAO models in rat and mouse pups [83,220,221]. After an MCAO stroke in P9 mice, there was no increase in microglia (CD11b^+^, CD45^intermediate^) at three days post-stroke, but CD11b^+^, CD45^high^ monocytes were substantially increased [221]. Similarly, Chen and colleagues [222] observed a substantial influx of monocytes (CD45^high^, CD11b^+^) but no change in microglia number (CD45^intermediate^/CD11b^+^) at three days post-injury after permanent MCAO (pMCAO) in P16 rats. Interestingly, in the P9 mouse MCAO model, while no difference in lesion volume, cell death, or microglia number was observed between males and females, CD11b-expressing cells from males showed more robust pro-inflammatory gene expression and monocyte infiltration (CD45^high^/CD11b^+^) [221]. This suggests that while microglia numbers may not differ significantly, there are substantial sex-specific functional differences. Nevertheless, significant gaps remain in our understanding of microglial roles in neonatal stroke due to limitations in previous tools and techniques, poor reporting on multiple aspects of phenotype, and a focus on simple counts. Thus, it remains unclear how microglia either injure or protect the neonatal brain and whether all microglia or only specific subpopulations possess endogenous protective functions.

#### 3.5.2. Astrocytes

Astrocytes regulate extracellular concentrations of neurotransmitters and ions, regulate the integrity and activity at the blood–brain barrier (BBB, see section below), and secrete neurotrophic factors that support neuronal growth and function [223]. As a result of hypoxic stress, ATP depletion reduces astrocyte glutamate uptake, increasing extrasynaptic glutamate and leading to excitotoxic cell death [224]. Following injury and immune challenges, astrocytes undergo a process of ‘activation’ called astrogliosis, where they increase the production of glial fibrillary acidic protein (GFAP) and produce pro-inflammatory cytokines such as IL-1β, IL-6, and TNF-α, which can reduce the integrity of the BBB [225]. Like microglia, the immune activation of astrocytes has a simplified nomenclature, one that includes the ability to acquire a classically pro-inflammatory response, termed an A1 phenotype, or a classically anti-inflammatory or immunomodulatory phenotype, termed A2. These phenotypes are associated with outcomes in the developing brain after injury [226,227].

Immune-reactive astrocytes also increase aquaporin-4 expression, which is linked to oedema formation, increasing intracranial pressure, and impaired blood flow and nutrient availability [228]. According to Vizuete et al. [229], increased AQP4 mRNA expression in response to BBB damage caused by a striatal excitotoxic lesion in the adult rat strongly suggests it is crucial in re-establishing brain osmotic equilibrium post-oedema. Similarly, increased AQP4 expression on astrocyte end-feet in 10-day-old rat pups undergoing transient blockage of the MCA [230] led the authors to conclude that agents stimulating AQP4 expression could aid in clearing water from the brain parenchyma, potentially enhancing neurological outcomes in neonates after an HI insult. AQP4 modulation as a therapy would be particularly relevant in newborns, as their brain water content is significantly higher than in later stages of life. AQP4 mRNA knockdown in neonatal piglets subjected to cerebral HI (carotid artery ligation and hypoxia exposure at 3–5 days of age) protected against cytotoxic oedema, resulting in smaller infarct volumes and significantly improved neurobehavioral outcomes [231]. However, as cytotoxic oedema occurs within 24 h of injury [230], treatments aimed at reducing AQP4 levels soon after ischemia are impractical in newborns with a delayed stroke diagnosis.

Astrocytes produce extracellular matrix proteins during chronic injury stages, forming a glial scar with negative and positive effects. Astrocytes in the scar regulate cerebral blood flow, modulate immune activities, and stimulate neuroregeneration [232]. They also produce growth inhibitory molecules such as laminin, fibronectin, tenascin C, and proteoglycans (reviewed in detail [233,234]). Astrocytes also produce hyaluronic acid, the fragments of which can activate Toll-like receptor TLR2/4 and CD44 receptors, driving inflammation and further impairing the BBB [235,236,237]. Liu and colleagues induced cortical infarction using photothrombosis in adult wild-type (WT) and GFAP^−/−^ Vimentin^−/−^ mice [238,239]. The GFAP^−/−^ Vimentin^−/−^ mice developed a smaller scar and lower chondroitin sulphate proteoglycan (CSPG) distribution in the lesion boundary zone, which correlated with impaired functional recovery but elevated CSPG levels in regions remote to the injury. Different subclasses of CSPGs, such as NG2, brevican, and phosphocan, can promote or inhibit axonal regeneration [240]. Liu’s previous study also linked functional recovery at seven days post-MCAO with local axonal sprouting and outgrowth within the denervated spinal grey matter rather than long-distance axonal regeneration from the brain [238]. This indicates that reprogramming astrocyte function and CSPG production, rather than ablation, will most effectively enable astrocyte-directed neuronal regeneration.

The glial scar differs in the adult and neonatal brain, as demonstrated by Teo and colleagues using the endothelin-1 model of focal ischemic stroke in adult and infant non-human primates (marmoset, *Callithrix jacchus*) [24,45]. While lesion territory (i.e., the proportion of cortex impacted) was comparable between animals injured at 21 days and one year of age, the GFAP-reactive scar volume and neuronal loss were substantially smaller in neonates compared to adults. Interestingly, although the loss of neurons in neonates was more severe in the peri-infarct region (600 μm from the lesion), the severity of damage decreased quickly and was almost absent at 800 μm from the lesion. In comparison, neuronal loss extended to 2000 μm from the lesion core in adults [45].

### 3.6. The Blood–Brain Barrier

The BBB consists of a physical barrier formed by a layer of brain vascular endothelial cells with tight junctions, a capillary basement membrane, pericytes, and astrocyte end-feet. It also includes a transport barrier with membrane enzymes, transporters, and vesicular mechanisms [241,242]. The BBB selectively regulates substance transport between the blood and brain by controlling the expression by endothelial cells, pericytes, and astrocytes of a variety of tight junction proteins and efflux transporters [243]. The BBB forms early in foetal life with endothelial cells and pericyte recruitment into the brain, preceding astrocyte generation [244,245,246]. In rats, the first vessels develop in the cerebral cortex around embryonic day (E) 11, whereas astrogliogenesis occurs closer to birth [247]. However, the exact timing varies among species and regions. The functional abilities of the BBB at birth in humans and other mammals are a subject of ongoing study [248]. However, the notion that the neonatal barrier is dysfunctional has been described as a myth [249]. Indeed, the physical function of the BBB is similar in neonates and adults, and the activity of its various transporters is dynamically modulated across development to meet the brain’s specific needs [250,251].

Oxidative and cytokine-induced endothelial cell damage, including dysfunction of endothelial tight junction proteins, causes structural disruption of the BBB (reviewed in [252]). HI-induced ROS, lipid peroxides, peroxynitrite, and hydroxyl radicals contribute to BBB injury in acute neonatal ischemic stroke [233], increasing paracellular permeability and facilitating immune cell infiltration. Benjelloun and colleagues [253] were among the first to demonstrate that BBB disruption is followed by gliosis and leukocyte infiltration, inducing an intense local inflammatory response after permanent MCAO in P7 rats. Endothelial cells and peripheral leukocyte interaction are also key in disrupting the BBB. Neutrophil infiltration contributes to BBB disruption following transient cerebral ischemia in adult humans and adult stroke models [29,137]. However, in contrast, there is limited neutrophil infiltration into neonatal brains after transient MCAO [29] and very limited [30] or brief [254] infiltration into HI-injured neonatal rat brains. The mechanisms limiting neutrophil infiltration in neonates are not fully understood, but the varying expression of adhesion molecules, matrix metalloproteinases, and shifting chemokine gradients may play a role [29]. Despite being lower in overall number, inducing neutropenia or inhibiting leukocyte adhesion reduces injury in neonatal stroke models (P7, MCAO) [29], as reviewed in detail by Mülling [255], indicating an essential role for these cells.

BBB breakdown also causes vasogenic oedema due to the extravasation of high-molecular-weight molecules and water into the brain, which causes injury by driving post-ischemic inflammation [256,257]. The BBB response to stroke differs between adults and neonates: adult rats at 24 h post-MCAO show increased leakage of intravenously administered fluorescently labelled albumin (65 kDa) and small and large intravascular tracers (3 and 70 kDa), but neonates subjected to a similar insult do not [29].

Disrupted pericyte–endothelial cell interactions drive neuroinflammation after CNS injury and disease [258]. Pericytes form a lock-and-socket junction with endothelial cells to maintain the integrity of the BBB; they also guide the expansion of endothelial cells after birth by regulating the expression of Mfsd2a, a protein that inhibits transcytosis in endothelial cells [259]. Transcriptional profiling of endothelial cells, isolated 24 h after MCAO in adults and neonates, shows that the neonatal BBB has higher resilience to stroke than the adult BBB. Neonates more effectively preserve the gene expression and protein levels of tight junction and basement membrane components, such as collagen IV and laminin [29]. Additionally, BBB disruption is greater in 21-day-old rats compared to 2-h-old pups after a local inflammatory challenge, such as intrastriatal IL-1β injection [260].

In the immature brain, poor glutamate clearance from the brain to the blood increases the vulnerability of the BBB and neurons in the developing cortex to excitotoxic damage [261]. These developmental differences in clearance are driven by lower expression of the main driver of glutamate uptake, excitatory amino acid transporter 2 (EAAT2), in foetal and neonatal life [262]. Proteomic analyses reveal that neonates preferentially express proteins related to glycolysis, whereas adults have more aerobic oxidative enzymes, which may provide greater resistance to HI. Immature microvessels have fewer alternatives to glycolysis for their energy supply, making them more susceptible to vascular damage during times of high energy demand in the developing brain [263]. These findings indicate that developing brain endothelial cells may be more sensitive to HI, highlighting the need for multimodal approaches and age-specific analysis in translational models [264].

### 3.7. Kynurenine Pathway Dysfunction

The kynurenine pathway (KP) is the major catabolic pathway for the metabolism of tryptophan, an essential amino acid, into biologically active metabolites such as kynurenine, kynurenic acid (KYNA), 3-hydroxykynurenine, quinolinic acid (QA), 3-hydroxyanthralinic acid, anthranilic acid, and picolinic acid [265]. KYNA is both neuroprotective and anti-inflammatory, while QA is neurotoxic and pro-inflammatory [266]. Neurons, astrocytes (which primarily produce KYNA), and microglia (which predominantly generate QA) contribute to KP metabolite production, influencing neurotransmission and inflammation [173,265]. These metabolites play a pivotal role in neuroprotection, neurotoxicity, and immune modulation, thereby influencing brain health and disease [267].

Although the role of the KP in infants with stroke is not clear, there is evidence that it plays a role in injury in infants exposed to global HI. For instance, QA was increased in the cerebrospinal fluid of asphyxiated infants [268]. QA can induce a pro-inflammatory state in microglia and damage neurons by agonist effects on NMDA receptors, as well as induce ROS [269]. Conversely, neuroprotective kynurenine and KYNA were decreased in a piglet model of HIE, induced by ventilation of the piglet for 20 min with 6% O_2_ and 20% CO_2_, but increased by therapeutic hypothermia, the standard of care for term-born infants diagnosed with HIE [270]. Of interest, KYNA production is higher in foetal sheep brains compared to newborn and adult sheep brains [271], with reduced levels in the hypothalamus and hippocampus following HI injury induced by umbilical cord embolisation, suggesting a heightened susceptibility to acute hypoxia or ischemia in these areas linked to the KP [271].

In neonatal stroke models, the KP modulates glial cell function, influencing the progression and outcome of cerebral ischemia [272]. Preclinical evidence suggests that therapies involving kynurenine and kynurenine-3-monooxygenase (KMO) inhibitors could be effective neuroprotective strategies, potentially diminishing infarct size and improving behavioural outcomes [273]. Increasing brain concentrations of KYNA through L-kynurenine administration or probenecid provides neuroprotection in 7-day-old neonatal rats exposed to HI induced by carotid artery ligation + hypoxia or excitotoxic (NMDA-induced) lesions [274]. Increasing KYNA after HI in 7-day-old neonatal rats (induced by carotid artery ligation) reduced lesion severity, improved the excitatory amino acid profile at two weeks [275], and preserved neurons in the hippocampus and cortex associated with notably decreased levels of ROS, glutathione, and antioxidant enzyme activity [276].

### 3.8. Sex as an Independent Risk Factor for Neonatal Stroke

Male sex is a significant risk factor for the occurrence and severity of perinatal brain injury in humans and preclinical animal models, including encephalopathy of prematurity, HIE, and perinatal stroke. In neonates presenting with stroke symptoms, male infants show earlier signs of distress [277], and in rodent models, seizures are more frequent in males [278]. The underlying cause of male susceptibility is not well understood, but it is likely to involve hormonal influence [279,280] and other differential gene expression patterns [281], including those related to cell death and stress pathways.

Du and colleagues found that 16–18-day-old male rats are more sensitive to nitrosative cellular stress and excitotoxicity, perhaps due to lower brain glutathione peroxidase activity than females [282]. Female rats also exhibited higher activity of mitochondrial glutathione, the primary enzyme responsible for antioxidant defence against ROS, than males. These events are significant in Parkinson’s disease, traumatic brain injury, and cerebral ischemia, which are all more prevalent or severe in males compared to females.

After unilateral HI in P9 C57/BL6 mice, there was greater translocation of apoptosis-inducing factor from the mitochondria to the nucleus and reduced caspase 3 cleavage in male mice than in females [283]. Similarly, after unilateral focal ischemia in 7-day-old rats, females had 4-fold higher levels of caspase 3 cleavage than males [152]. Hypoxia (10.5% O_2_) from E15 to E21 in mice at P7 induced a sexually dimorphic gene expression signature, including increased NF-κB in oligodendrocyte precursors, astrocytes, and microglia in males [281].

During development, hippocampal microglia in female mice mature to a homeostatic state faster than in male mice, based on gene expression profiling, and a neonatal endotoxin immune challenge accelerates male microglia maturation to match that of females [284]. In an adult mouse permanent MCAO study, injury was worse in males but was mitigated by replacing male microglia with female microglia [285]. This suggests that male microglia allow for a greater degree of injury, possibly linked to a more classically pro-inflammatory state than female microglia, as recently reviewed [286]. Post-mortem studies reveal an increased number of microglia in infants with severe hypoxic-ischemic (HI) injury [287]. However, detailed phenotyping has not been undertaken to assess the expression of morphology and the multiple proteins indicative of reactivity. Additional analysis is required, as microglia are not necessarily harmful, with some subpopulations playing a positive protective role. Early studies in a rat MCAO model of neonatal stroke revealed that microglial ablation worsened injury, highlighting their protective effects even in the acute phase of injury [217,218,288].

## 4. Current Therapeutic Options for Reducing Tissue Injury or Repairing Brain Damage After Neonatal Stroke

**Figure 4:**
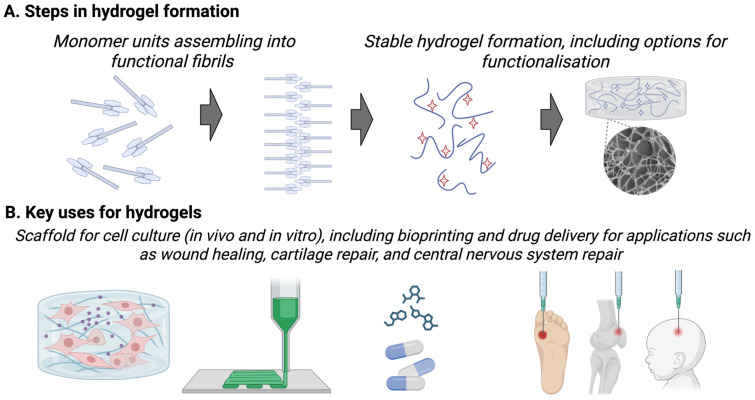
(**A**) A simplified representation of the generation of a hydrogel from monomer units assembling into functional fibrils and then the formation of a stable hydrogel, including the option to functionalise the hydrogel with molecules (red stars). (**B**) An indication of the uses of hydrogels, such as for 3D cell culture, bioprinting into biosensors, drug delivery, and for tissue engineering including for wound healing, cartilage repair, and central nervous system repair.

Knowledge regarding treatment options for neonatal stroke comes from stroke-specific research and also studies testing treatments designed for infants presenting with encephalopathy with symptoms suggestive of hypoxia-ischaemia (i.e., HIE). Within this HIE group, a small number of infants will experience stroke, be it arterial or cerebral sinovenous thrombosis. We can learn from these studies, but we must acknowledge that the treatments were not explicitly developed for stroke, which has a different aetiology and injury pattern. Most therapies under development are systemically delivered, necessitating the careful evaluation of safety across organs and organ development. We will discuss below a handful of treatments, including those used in adults (recombinant tissue plasminogen activator), those already in early clinical trials (stem cells) or ‘accidentally’ used off-label (hypothermia), those with established preclinical therapeutic efficacy but pending issues (melatonin), and experimental stage therapies (growth factors and neurotherapeutic peptides). A novel approach is to deliver therapies directly into the injury site, and we will discuss this implant approach, mediated by injectable biomaterials, i.e., hydrogels (Figure 4).

### 4.1. Recombinant Tissue Plasminogen Activator

Tissue plasminogen activators, such as alteplase, are trypsin-like serine proteases that convert plasminogen to its active form, plasmin [289], to effect clot dissolution. They can be effective for treating adult ischemic stroke if administered within 4.5 h of stroke onset [290,291,292]. tPA is contraindicated for haemorrhagic stroke due to the danger of haemorrhagic conversion for ischemic stroke patients, resulting in it being used for only ~5% of adult stroke patients [293]. However, it is expected that with increased due diligence and expansion of the tPA therapeutic window (from 3 to 4.5 h), rates will increase. However, its use in infants is limited due to the delay in diagnosis and age-related haemorrhage risks, making it unsuitable in neonates [10,294]. The low recurrence rate after NAIS and the lack of association with thrombophilic factors such as cysteine, lupus anticoagulant, and blood protein C/S deficiency further discourage the routine use of drugs targeting coagulant processes, which are common adult treatments [295].

### 4.2. Stem Cells

Stem cell therapy has proved to be an effective treatment for reducing brain damage in up to 80% of preclinical models of neonatal brain injury; these treatments include neural stem cells, mesenchymal stem cells (MSCs), human umbilical cord blood cells, hematopoietic stem cells, and stimulation of endogenous stem cell production (reviewed recently [296,297,298,299,300,301]). The PASSIoN trial is the first-in-human trial for treating neonatal stroke; it reported positive safety data in Phase 1 [302] and is due to progress to a Phase 2 trial. A 2023 Cochrane review highlighted that no other trials had been conducted, and none were registered to directly support the efficacy of stem cells for neonatal stroke [303]. Stem cells are being tested in term-born infants with HIE across multiple countries, with excellent safety profiles, and several studies have reported positive early Phase 2 data [304]. As such, stem cell treatments show great promise to become an important therapeutic option for neonatal stroke in the next five to ten years based on positive phase I and Phase II trials for neonatal stroke, HIE, and intraventricular haemorrhage [302,305,306,307,308]. However, it will be a challenge to deliver these therapies to every infant due to cost and resource availability, and some individuals will be unwilling to undergo such treatments. Therefore, exploring alternative therapeutic options remains important.

### 4.3. Hypothermia

In clinical practice, hypothermia involves cooling the neonate’s brain to 33 °C for three days. Hypothermia must be initiated within 6 h of birth and is only partially effective for treating HIE [309,310]. The safety of hypothermia in stroke patients has been determined ‘by accident’ as part of studies of HIE, as distinguishing between infants with HIE and stroke is difficult without imaging [311]. As such, a small proportion of infants in any HIE cohort are retrospectively identified as having stroke. In one such study of five infants with stroke treated with hypothermia, none had seizure activity, compared with the seizures observed in 7/10 non-treated stroke-affected infants [312]. However, long-term outcomes have not yet been reported. The difficulties of using hypothermia in the stroke population have been thoroughly reviewed elsewhere [313], but include shivering control, airway management (i.e., the need to intubate as infants are commonly sedated during hypothermia), and prevention of complications. Studies of hypothermia for neonatal stroke have predominantly been powered to assess feasibility and safety, but increased complications or severe negative outcomes have been reported [314]. Hypothermia is effective in a rat model of neonatal stroke, but in these models, the issues of intubation and cardiac stability are not present [315]. The main problem with wider implementation is the typical delay in diagnosis of neonatal stroke (over 22 h on average). As such, there is a great need to design treatments that prevent delayed, tertiary-phase injury processes [136,146], stimulate neurorepair and regeneration, and are compatible with hypothermia as the currently accepted standard of treatment.

### 4.4. Melatonin and the Tricky Excipient—Ethanol

Melatonin, with its anti-apoptotic and anti-inflammatory effects, has an excellent safety profile and striking abilities to prevent and treat HI-associated brain injury across species [316,317,318,319,320], including in a model of neonatal stroke [321]. Melatonin has an oral bioavailability of around 5% in adults [322], although this availability is higher in newborn infants in practice due to slower hepatic and renal clearance [323]. A stable, ethanol-free intravenous formulation that facilitates the clinical use of melatonin remains elusive, despite decades of research and development. One study is of interest in the hunt for an appropriate excipient for melatonin. In a piglet model of term infant HIE, when combined with hypothermia, melatonin treatment (18 mg/kg in 2.5% ethanol excipient over 2 h) was strongly neuroprotective compared to the ethanol-only plus hypothermia control treatment [324]. Notably, the ethanol-only plus hypothermia control was neuroprotective compared to the saline-only plus hypothermia control—i.e., the ethanol was neuroprotective. Specifically, melatonin and the ethanol-only treatments combined with hypothermia were associated with accelerated amplitude-integrated electroencephalography (aEEG) recovery and reduced cell death. Additionally, both treatments were linked to increased oligodendrocyte survival compared to the hypothermia–saline-treated group. Ethanol has also been shown to be neuroprotective in adult models of HI injury [325,326], supported by large epidemiological studies of stroke risk in adults [327,328].

In the first study [324], the blood alcohol concentration (BAC) was not reported. However, this dose would likely exceed the European Medicines Agency’s conservative threshold of 2.5 mg/dL, although it would not exceed the threshold of 25 mg/dL from the American Academy of Paediatrics. In a subsequent study, Pang et al. (2024) [319] used a higher dose of melatonin and higher ethanol concentration for much longer to achieve even more robust neuroprotection in the piglet HI model: 20 mg/kg in 5% ethanol over 2 h; then 10 mg/kg in 5% ethanol every 12 h between 24 and 60 h. This treatment resulted in a peak BAC of below 25 mg/dL at three hours and maintenance levels from 2 to 7 mg/dL. It is worth comparing these levels to common ethanol doses used to model foetal alcohol spectrum disorders (FASD), in which 2.2 to 5.0 g/kg of ethanol typically results in BACS of 100–400 mg/dL over multiple days, with the greatest risk in the period equivalent to the first trimester in humans [329,330,331,332,333]. Thus, although ethanol is not the first choice for an excipient; the evidence supporting melatonin—and our current use of other medications requiring low-dose ethanol [334] when the benefits outweighs the harms—suggests that more preclinical work is justifiable to understand any potential for melatonin used with an ethanol excipient as a neuroprotectant.

The precise mechanisms underlying ethanol-induced neuroprotection are not understood, but could result from its modulation of GABAergic neurotransmission [335]. Signalling via GABA is inhibitory in the mature brain but excitatory in the immature brain [336]. Specifically in piglets, this developmental shift in GABAergic signalling is reflected by a peak in excitatory GABAA receptor α3 subunit expression at 100 days gestation (term = 114 days), followed by a gradual decline. In contrast, inhibitory α1 subunit levels rise after birth and peak by postnatal day 7 [337]. In addition, ethanol has been shown to attenuate hyperglycolysis and suppress NADPH oxidase activation following ischemic injury, thereby reducing oxidative stress and metabolic dysfunction in an adult model [338]. However, ethanol has also been shown to enhance TLR signalling and increase pro-inflammatory cytokine levels following high-dose exposure in adolescent rats, but not in adult rats [339]. The effects in even less mature brains remain unknown. Many drugs impacting GABAergic signalling are our only options to treat disorders and injuries in infants (such as seizure medications and anaesthetics). Thus, it is surprising that our knowledge of the specific timing, spatial pattern, and regulation of the switch from excitatory to inhibitory GABA signalling in humans is poorly understood [80,81,340]. Knowledge of this process is essential for understanding the effects of ethanol on the developing brain and the potential therapeutic utility of melatonin in ethanol-induced injury as a treatment for infants with HI-associated conditions. As for the hydrogel treatment to be described below, this approach may be valuable for infants with the most severe injury (optimal risk-benefit relationship) and as a delayed therapy where the inhibitory action of GABA can be more clearly established.

### 4.5. Growth Factors and Neurotherapeutic Peptides

An attractive alternative to the complexity of delivering live cells to a patient is to use isolates of extrinsic factors, including vascular endothelial growth factor (VEGF), BDNF, granulocyte-colony stimulating factor (G-CSF), erythropoietin (EPO), and neurotherapeutic peptides [168,341,342]}. However, systemic delivery can lead to issues with off-target effects and the short half-lives of recombinant growth factors may limit their impact [343]. Advances in chemical engineering are addressing the challenges of physiologically short half-lives, off-target effects, and the inability to cross the blood–brain barrier (BBB) [344,345]. Directly delivering therapeutic agents to the brain using biocompatible hydrogels might mitigate these issues [346], as discussed in more detail below.

## 5. Hydrogels

Hydrogels are water-swollen polymer chains linked by physical or covalent bonds [347] forming an entangled or cross-linked network. They can be comprised of natural polymers such as laminin, collagen, or hyaluronic acid, semi-synthetic polymers, such as self-assembling peptides (SAPs), and fully synthetic polymers, such as PEG-DA (Figure 4) [348,349]. Hydrogels possess several properties that make them appealing candidates for treating neonatal stroke, including physical characteristics that allow delivery via a small cannula needle (shear thinning properties), the ability to carry multiple neurotherapeutics into specific loci in the brain in a stimulus-sensitive and time-dependent manner, physical support to prevent cyst formation, controlled degradation, and the presentation of extracellular matrix (ECM) motifs to stimulate regeneration [350].

Two different properties allow hydrogels to be delivered into the brain through small-bore delivery devices (e.g., a needle), and then ‘re-gelate’ in situ for targeted treatment [351]. Firstly, hydrogels can have shear-thinning properties, where the viscosity decreases as the shear rate (or the rate of deformation) increases. Injectable hydrogels made from self-assembling peptide structures are a versatile potential treatment for neonatal stroke because they are capable of mimicking CNS tissue properties [352,353], can deliver bioactive agents such as growth factors, peptides, and stem cells directly into the injured tissue to protect against further toxic damage, and can also regenerate the lost tissue in the brain [354,355,356,357]. In addition, polymers such as poly(*N*-isopropylacrylamide) (PNIPAM) undergo a reversible change in structure and solubility at their lower critical solution temperature (LCST), resulting in a temperature-dependent volume phase transition. For instance, when at or below its LCST of 32 °C—i.e., slightly below human body temperature (37 °C)—PNIPAM is a liquid that can be delivered via a small-gauge needle, after which it polymerises into a gel inside the body. This approach has been employed to deliver miR-138-modified stem cell exosomes via an injectable polyglycolic acid copolymer and polyethylene glycol (PLGA-PEG-PLGA) hydrogel to the site of injury in a spinal cord injury (SCI) model using 10-week-old female Sprague Dawley rats. This hydrogel preparation released approximately 80% of the encapsulated exosomes within three days [358], facilitated functional neurological recovery, and significantly reduced TNF-α, IL-1β, and IL-6 levels compared to the untreated SCI group seven days post-injury.

In practice, delivering a hydrogel into the infarct core would involve imaging-guided needle insertion. This is invasive, but less so than current well-established approaches for the treatment of hydrocephalus linked to intraventricular haemorrhage (IVH), myelomeningocele, and meningitis [359,360]. In addition, the technology for neurosurgical delivery of agents into the brain, i.e., convection-enhanced delivery, has matured in the adult field and is used regularly to deliver treatments for glioblastoma [361], as have approaches for implantation of deep brain stimulation devices [362].

In addition, hydrogels can be engineered so that the release of therapeutic agents is temporally ‘tuned’ or made stimulus-sensitive; these hydrogel adaptations optimise their positive impacts on regeneration and repair. Release tuning is mediated by physical entrapment, chemical conjugation, and sensitivity to magnetic fields, temperature, pH, and light. Below we outline examples of hydrogels for the treatment of severe focal brain injuries that have characteristics of interest for treating neonatal stroke.

Physical entrapment involves directly mixing drug molecules with the hydrogel precursor before cross-linking to form the hydrogel, a process known as co-assembly. This approach allows for control of the drug release rate through the manipulation of the hydrogel mesh size and degradation rate. Hydrogel degradation refers to the hydrolysis of the cross-links or the polymer backbone over time, which is crucial for drug delivery applications. Polymer-based hydrogels protect encapsulated drugs from degradation or inactivation by shielding them from destabilising processes in the body, such as enzymatic degradation and pH variations, preserving their stability and therapeutic activity. Controlled biodegradability ensures safe metabolism and elimination from the body, with a degradation rate tailored to match desired drug release kinetics and the tissue healing timeline [363].

The physical entrapment of glial cell line-derived neurotrophic factor (GDNF) within fluorenylmethoxycarbonyl-aspartic acid-isoleucine-lysine-valine-alanine-valine (Fmoc-DIKVAV) hydrogels has been used for neural transplants in Parkinson’s disease models, improving graft cell survival, host tissue reinnervation, and tissue repair [356]. The hydrogel stabilises GDNF, allowing for a gradual release that peaks at 12 h and reaches 57% at 48 h, compared to release within one hour for soluble GDNF. This controlled release is particularly attractive if hydrogels are used to regenerate large brain injuries, which may take many months or even years.

In a more complicated entrapment approach, Wang and colleagues [364] encapsulated EPO and epidermal growth factor (EGF) that was modified via PEGylation to increase solubility and stability (EGF-PEG) in PLGA nanoparticles. This was undertaken using biphasic microparticles, in which the two phases have different physical or chemical properties. These EGF-PEG/EPO polymeric particles were administered four days after inducing a stroke-like lesion in 9–11-week-old male mice using cortical endothelin-1 injection, a clinically relevant time point of delivery. Treatment decreased the number of GFAP^+^-reactive astrocytes and CD68^+^ pro-inflammatory macrophages and microglia, and increased neural stem/progenitor cell proliferation, expression of antigen Kiel 67 (Ki67), and neuronal nuclear antigen (NeuN) positive cells. The treatment also reduced cavity size and enhanced neural stem/progenitor cell proliferation in the subventricular zone 18 days post-treatment. This timing and the ability to stimulate endogenous stem cell proliferation hold promise for neonatal stroke therapy, which is crucial for regenerating substantial lesions in infants who have the poorest outcomes due to neonatal stroke.

pH-sensitive hydrogels include polymers such as polyacrylic acid (PAA), which undergo protonation and deprotonation in response to changes in pH, leading to changes in swelling and charge density. The pH in the infarct region is approximately 6.5, compared to 7.0–7.4 in uninjured brain regions [113,114], making pH-sensitive drug release from a hydrogel relevant for stroke. In a study using an adult rat model of MCAO to simulate stroke, pH-sensitive polymeric micelles loaded with lysozyme (lysozyme-pH-PM) and SDF-1α (SDF-1α-pH-PM) were administered intravenously and effectively delivered the target proteins to the ischaemic site. There was an increase in vascular density in the ischemic cortex of rats following intracerebral administration of SDF-1α, promoting neurogenesis and angiogenesis [365]. As decreased cerebral pH persists in the neonatal brain for weeks after HI injury [115,116], this approach may be highly relevant to neonatal stroke, where any treatment would be delayed due to diagnostic issues and the need for neurosurgical planning.

The SAP Fmoc-DIKVAV hydrogel, composed of fluorenylmethoxycarbonyl (Fmoc)-protected laminin-based epitopes, has shear-thinning properties and a nanofibrillar structure (23–38 nm) that resembles natural ECM proteins such as collagen (20–200 nm). It also self-assembles with isoleucine-lysine-valine-alanine-valine (IKVAV) on the outermost layer [366] into a nanofibrous network that mimics laminin and mediates neurite outgrowth, facilitating neural migration and regeneration [366,367]. Co-assembly of the Fmoc-DIKVAV hydrogel with fucoidan, a potent anti-inflammatory and antioxidant polysaccharide, further enhances its regenerative efficacy, increasing its ability to attenuate astrocytic reactivity (GFAP^+^ cells) in a rat model of TBI [366]. Additionally, Fmoc-DIKVAV serves as a drug delivery system, preventing peptide degradation and enabling controlled, temporal release of the agent via various encapsulation techniques [344,354,368].

Hydrogel research is working towards overcoming its current limitations. Firstly, hydrogels can be designed to be biodegradable and minimally immunogenic [369,370], but it is challenging to fully predict how the immature neonatal immune system may respond, potentially leading to neuroinflammation or altered healing responses. Furthermore, any mechanical mismatch between the hydrogel and developing brain tissue could impair integration and potentially worsen injury or disrupt neural circuit formation. Additionally, as the mechanical properties of the brain change with age, addressing the critical interaction between the degradation rate and mechanical properties will be essential. Approaches to model hydrogel characteristics in complex environments and engineer hydrogels that overcome these challenges are emerging to overcome these issues [371,372].

Furthermore, ethical and regulatory considerations introduce additional barriers to clinical implementation; however, as more clinical trials of bioengineered therapies are conducted, the approach of clinicians and regulatory bodies will evolve [373,374]. Long-term safety data are currently lacking. To overcome these issues, careful use of preclinical models will be necessary. However, we could identify only two models of neonatal stroke in a large animal species, the piglet [375] and non-human primate [45], that were tailored explicitly to neonatal stroke’s biological and developmental context.

## 6. Conclusions and Future Perspectives

Significant obstacles in perinatal brain injury trials include the selection of infants who may benefit from the trials, complicated by poor prognostic indicators for neonatal stroke, delays in diagnosis, and varying outcomes among infants with similar lesions. Prognosis is clearer for infants with small or large lesions, but moderate injuries remain unpredictable. Including infants in trials who would do well without treatment substantially impairs our ability to determine treatment efficacy, and up to 50% of infants who develop a stroke will have typical developmental trajectories. Advances in imaging, including bedside imaging [73,376,377], integrated workflows that combine vascular and parenchymal imaging [378], significant advances in follow-up technology [379], and the development of improved methods for assessing soluble markers of injury [380], including point-of-care diagnostics [381], will help improve the understanding of injury volume and outcomes, aiding in the identification of those who need treatment.

Furthermore, biomarkers of injury severity and type (haemorrhagic versus ischemic) are being validated, although much more is known in adults than neonates currently, as reviewed in [382,383,384]. For instance, in adults there is strong evidence for the utility of matrix metalloproteinase-9 (MMP-9) [385], an enzyme involved in ECM degradation and BBB integrity, to predict complications like haemorrhagic transformation [386]. In addition, D-dimer is an attractive biomarker [387], as it is only created when fibrin clots form and are then degraded, making it a specific marker of active coagulation and fibrinolysis [388]. In the neonate, the astrocytic calcium-binding protein S100β is elevated in stroke and other perinatal brain injuries such as HIE and IVH [389]. Similarly, elevated levels of Activin-A, involved in inflammation, have been found in preterm babies [390], in those with IVH [391], and in an adult model of stroke [392]. No single biomarker has been validated, and multi-marker or precision approaches using machine learning to incorporate multiple data types are likely to lead to the best predictive value in such heterogeneous conditions [393,394]. The push to be able to measure multiple potential biomarkers simultaneously with methodological and technological development is also driving the utility of biomarkers forward [380,395]. Decreasing reliance on clinical imaging will decrease the time needed for diagnosis and clinical care costs.

Due to the high numbers of infants diagnosed with presumed perinatal stroke, understanding how we can improve outcomes for these infants is essential. The tertiary phase of injury is when processes that prevent the brain from being repaired, or lead to new problems, are present weeks, months, or years after injury [136,146]. It has been shown that after perinatal injury, there are changes in brain metabolite levels and immune responses, as well as sensitisation to further insult, which persist for many years [116,396,397,398]. Delayed administration of therapies is highly effective at improving outcomes in preclinical models of neurological injury [399,400,401]. Thus, there is a need to ensure clear patient stratification and that time to plan neurosurgery is not a limiting factor in the translation of a hydrogel therapy.

Combination therapies are proposed as a viable way to develop approaches to improve outcomes, as they allow for complementary pathways to be targeted, including processes in different phases of injury. In adult stroke, for instance, the anti-platelet aggregation drug clopidogrel and the nonsteroidal anti-inflammatory drug acetylsalicylic have been proven to be more effective than acetylsalicylic acid alone for preventing transient ischemic attack and improving outcomes after stroke [402,403]. As hypothermia is the standard of care for HIE, adding a therapy in combination to increase efficacy is necessary when planning clinical trials; but clearly, the combination approach is not straightforward. Combining drugs with hypothermia to enhance therapeutic efficacy was detrimental in sheep [404] and piglet [405] models of HIE. Furthermore, since at least 2013, efforts have been made to combine stem cells with hypothermia to reduce the incidence of injury in models of cerebral palsy [406]. However, a recent small clinical trial has shown that in HIE, there are no additive positive effects of hypothermia and stem cell treatment [407].

In conclusion, infant and adult brains respond differently to stroke, necessitating age-specific research to understand responses and develop therapies. Optimistically, we are only a few years away from having a clinically approved stem cell treatment for neonatal stroke. Therapies such as injectable hydrogels need to be designed to address the needs of infants with large injuries who fail to respond to stem cells or are unable to receive them. Additional research into affordable and easily deployable therapies, such as melatonin, is highly desirable, as this approach is crucial for providing options in low-resource settings.

## Figures and Tables

**Table 3:** A summary of neonatal arterial stroke locations, incidence. and impacts.

Arterial Territory	Incidence	Brain Regions and General Functions Impacted
*Middle cerebral artery (MCA) territory*	80–90%	Cortex: Frontal, parietal, and temporal lobes influencing motor, sensory, language, cognitive, and attention functions. Deep structures: Basal ganglia and internal capsule impacting sensorimotor integration and muscle tone.
*Posterior cerebral artery (PCA) territory*	5–10%	Occipital lobe: Visual cortex, which may cause visual field deficits Thalamus: Can affect sensory processing and motor relay functions
*Anterior cerebral artery (ACA) territory*	1–5%	Medial frontal lobes: Affecting lower limb motor control Cingulate gyrus and corpus callosum: Possible impact on motivation and interhemispheric communication
*Vertebral, basilar, and posterior inferior cerebellar artery (PICA) or superior cerebellar artery (SCA) territories*	<1%	Focal infarcts in cerebellar hemispheres or vermis impacting motor control, tone, and learning

Table data from [58,62,63,64,65,66,67].

## Data Availability

The authors will make any materials used to create this review available on reasonable request.
